# Supplementary figures and images for: Theoretical Evaluation of Impact Characteristics of Wavy Graphene Sheets with Disclinations Formed by Origami and Kirigami
Source: Nanomaterials (Basel). 2022 Jan 27;12(3):436. doi: 10.3390/nano12030436 (PMC8838731; doi:10.3390/nano12030436)

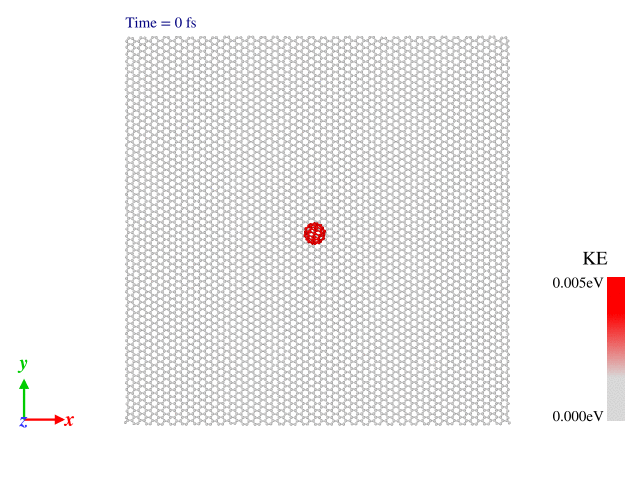

Supplement: Supplementary file 1 [file nanomaterials-12-00436-s001.zip › nanomaterials-1565475-supplementary/movie/idealGS.gif]

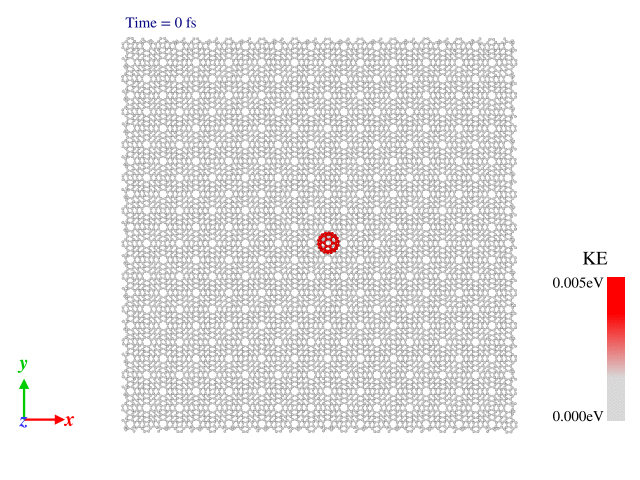

Supplement: Supplementary file 1 [file nanomaterials-12-00436-s001.zip › nanomaterials-1565475-supplementary/movie/wavy1.gif]

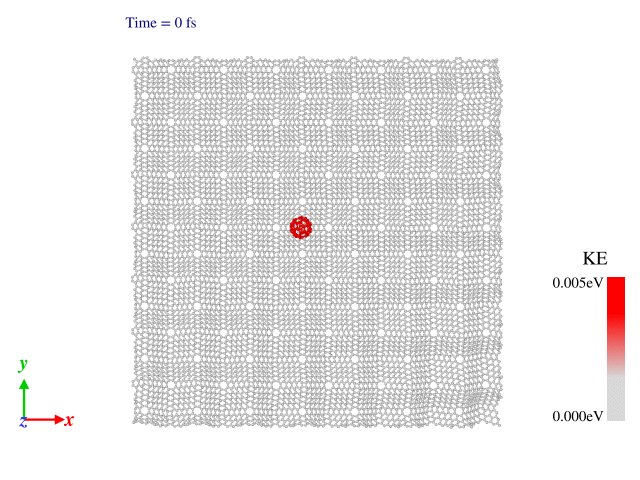

Supplement: Supplementary file 1 [file nanomaterials-12-00436-s001.zip › nanomaterials-1565475-supplementary/movie/wavy2.gif]

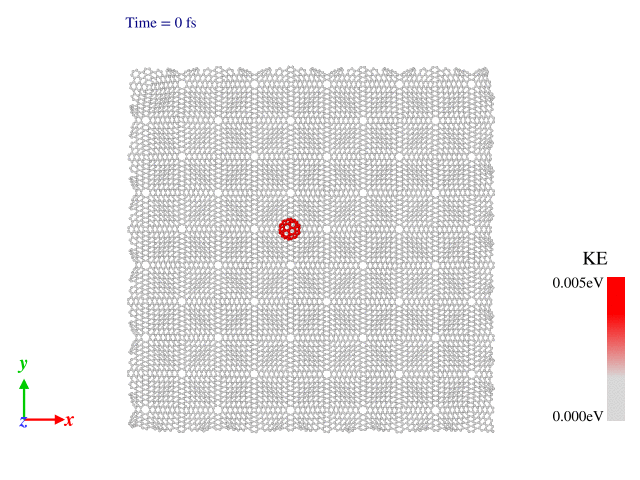

Supplement: Supplementary file 1 [file nanomaterials-12-00436-s001.zip › nanomaterials-1565475-supplementary/movie/wavy3.gif]

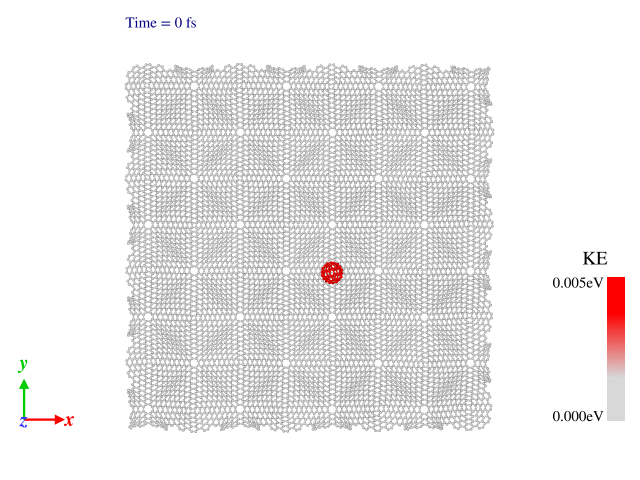

Supplement: Supplementary file 1 [file nanomaterials-12-00436-s001.zip › nanomaterials-1565475-supplementary/movie/wavy4.gif]
